# Do not treat ghosts: anti-methicillin-resistant *Staphylococcus aureus* (MRSA) therapy in osteomyelitis without identified MRSA

**DOI:** 10.1017/ash.2025.24

**Published:** 2025-02-17

**Authors:** Jincy Varughese, Annie Halfman, Matthew Crotty, Julie Alexander, Leigh Hunter, Mark Hupert, Edward Dominguez

**Affiliations:** 1 Department of Pharmacy, Methodist Dallas Medical Center, Dallas, TX, USA; 2 Department of Infectious Diseases, Methodist Dallas Medical Center, Dallas, TX, USA; 3 Department of Internal Medicine, Methodist Dallas Medical Center, Dallas, TX, USA; 4 The Liver Institute at Methodist Dallas, Methodist Dallas Medical Center, Dallas, TX, USA

## Abstract

**Objective:**

To compare the clinical outcomes of patients with lower limb osteomyelitis (LLOM) and negative methicillin-resistant *Staphylococcus aureus* (MRSA) cultures treated with anti-MRSA therapy (AMT) versus those treated with no-anti-MRSA therapy (NAMT).

**Design:**

Retrospective cohort study.

**Patients:**

Hospitalized adult (≥18 yr of age) patients admitted to multiple tertiary referral centers in a single healthcare system between April 1, 2017 and April 1, 2023, with LLOM and planned intravenous antibiotics for at least four weeks.

**Methods:**

Electronic medical records were queried for demographic information, admission dates, treatment strategies, imaging and culture results, and discharge diagnoses. Descriptive statistics measured baseline characteristics, imaging, and culture results.

**Results:**

Out of 473 patients, 64 met the inclusion criteria and 409 were excluded. Of the 64 patients, 26 (40%) had AMT and 38 (59%) had NAMT. A larger but statistically insignificant portion of patients in the NAMT cohort failed therapy (23% AMT vs 32% NAMT, *P* = 0.325). However, hospital readmission for LLOM within 180 days of antibiotic completion (46.2% vs 47%, *P* = 0.92), hospital length of stay (median (IQR): 6 (5–9) d vs 7 (5–12.5) d, *P* = 0.285), incidence of new renal replacement therapy initiation (0% vs 2.6%, *P* = 0.594), creatinine kinase levels (0 vs 2.6%, *P* = 0.594), and drug-induced immune thrombocytopenia (0% vs 5.3% *P* = 0.349) were comparable between the two cohorts.

**Conclusions:**

Treatment failure rates and adverse events did not differ significantly among patients with LLOM treated with AMT or NAMT. Further investigation of determinants of clinical failures in LLOM may help optimize overall treatment.

## Introduction

Osteomyelitis (OM) is an infection and inflammation of the bone or bone marrow, often due to an open wound, operation, or invasive trauma.^1^ There were ∼7.6 cases per 100,000 person-years in the United States from 2000 to 2009.^2^ OM is invasive and involves hematogenous seeding or contiguous spread of the infectious organism.^1–4^ As a result, it is associated with a high rate of relapse, large disease burden and steep health care costs.^4^ There is an economic burden in preventing or treating OM properly. For instance, Medicare spends $9 to $13 billion per year for inpatient costs, hospital admissions, and other expenses for those who have a diabetic foot infection (DFI), a common cause of OM.^5^ Due to the severity of infection, there is a lifetime lower extremity amputation incidence of 20%, a 5-year mortality risk of 50% to 70%, and a recurrence rate of 65% at 3 to 5 years in patients with OM.^6^

OM is classified by the location of the infection, extent of spread, chronicity, and source of infection.^4,6^ Common risk factors for OM include diabetes mellitus, peripheral vascular disease, immunosuppression, previous OM infection, intravenous (IV) drug use, and recent surgery. There are several organisms that can cause OM, but the most common pathogen is *Staphylococcus aureus*.^6,7^ Other common pathogens include *Pseudomonas*, *Streptococcus*, *Enterococcus*, and *Enterobacterales* species. Methicillin-resistant *S. aureus* (MRSA) is frequently identified in OM and is associated with an increased risk of recurrence, complications in treatment, and delay in recovery. Even with effective therapy, there is a 20% to 30% chance of clinical failure.^8^ *S. aureus* has adapted many strategies to improve resistance as it interacts with a host cell. Such methods include biofilm formation, toxin secretion, and small colony variant formation (ie, slow-growing bacterial subpopulations that can arise spontaneously in response to environmental stresses).^9^ These methods are a few of the driving forces that pilot the transition from an acute OM to a chronic OM infection. *S. aureus* also induces an inflammatory response, which initiates a host cell death by apoptosis and necrosis. Therefore, those with a immunosuppression can have worse outcomes and are more likely to develop OM.

Following confirmation of OM via imaging and histopathologic examination, treatment for OM consists of antibiotic therapy and often a surgical intervention.^3,7,10^ DFI-OM Guidelines by the Infectious Diseases Society of America/International Working Group on the Diabetic Foot recommends initially treating OM with broad-spectrum antibiotics that include coverage for *S. aureus.* Over time, antibiotic selection based on microbiology and antimicrobial susceptibilities should be narrowed to the specific pathogen grown on cultures. Treatment is then continued for four to six weeks if surgical intervention is not performed.^11^

As *S. aureus* causes 30% to 60% of OM cases, many patients receive therapies for both methicillin-susceptible *S. aureus* and MRSA even with negative MRSA culture data. It has become less common to receive therapy for the targeted pathogen.^12,13^ This practice is not new, as many studies have looked into this issue over the years. Unfortunately, there is limited evidence to support continuing anti-MRSA therapy (AMT) without microbiological evidence of MRSA. To address this gap in knowledge, a retrospective chart review was conducted to evaluate the clinical outcomes of patients with lower limb OM (LLOM) placed on definitive AMT, despite cultures negative for MRSA.

## Methods

### Study design

This multicenter, retrospective cohort study examined patients admitted to four acute care hospitals within the Methodist Health System in Dallas-Fort Worth, TX, USA between April 1, 2017, and April 1, 2023. The study compared the clinical outcomes of patients who did and did not remain on AMT while having cultures negative for MRSA. All data were retrieved using electronic health records and electronic medication administration records (Epic®, Epic Systems, Verona, WI). This study was reviewed and approved by the Methodist Health System institutional review board.

### Study population

**Figure 1.**
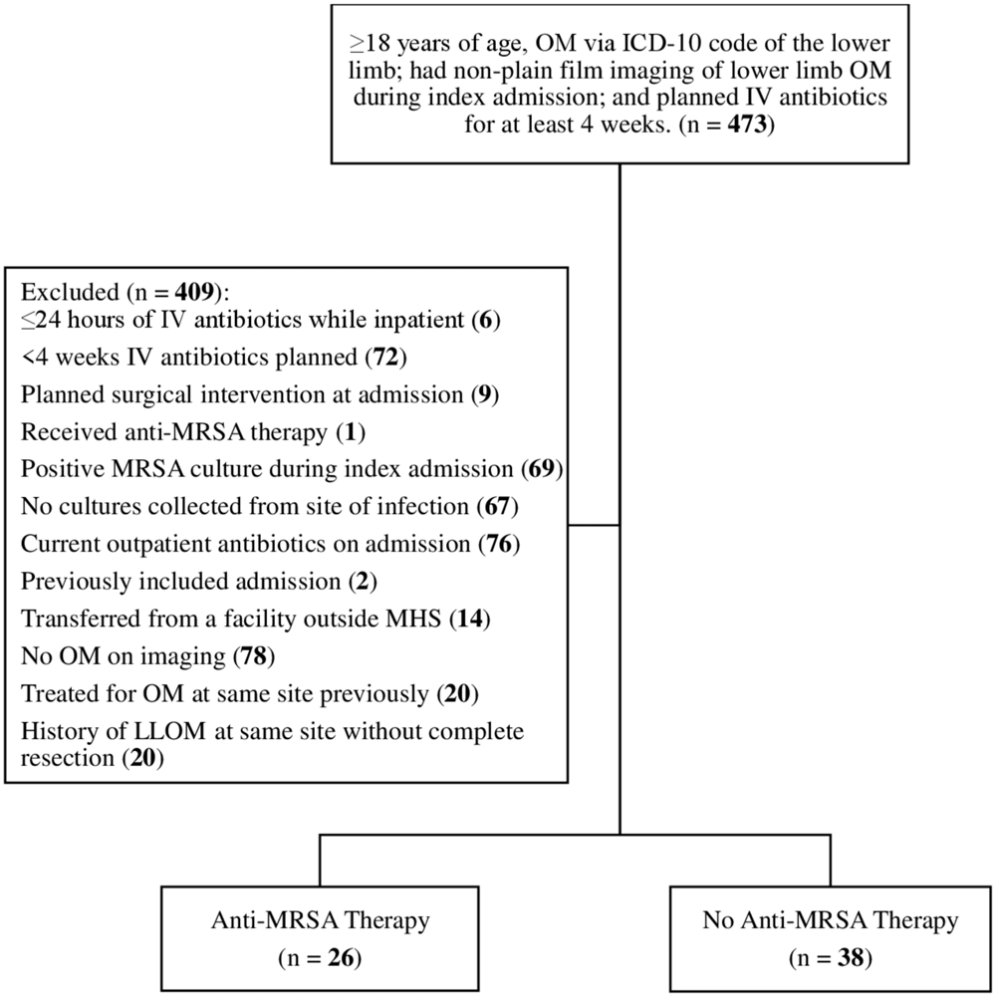
Flow diagram of the patient cohort. Abbreviations: ICD-10, International Statistical Classification of Diseases and Related Health Problems, 10th revision; IV, intravenous; LLOM, lower limb osteomyelitis; MHS, Methodist Health System; MRSA, methicillin-resistant *Staphylococcus aureus*; OM, osteomyelitis.

Adults (≥18 yr of age) were eligible if they had an ICD-10 code indicative of a LLOM, imaging (computed tomography, magnetic resonance imaging or x-ray) of LLOM during index admission and had plans for IV antibiotics for ≥4 weeks (Figure 1). Patients were excluded if they received IV antibiotics for ≤24 hours, had a planned surgical intervention documented at index admission, received a monotherapy AMT, had a positive MRSA culture during index admission, no cultures collected from site of infection (blood or wound), was on an outpatient antibiotic at index admission, additional admissions were excluded (patient included only once in the study), transferred from a facility outside of Methodist Health System and had a history of LLOM at the same site without a complete resection.

### Outcomes

The primary outcome of this study was a composite of treatment failure, which included repeat antibiotic treatment within 180 days of completion; unplanned surgical intervention during index admission after completing ≥4 weeks of antibiotic therapy; and death by any cause after ≥4 weeks of antibiotics during index admission. Secondary outcomes focused on rates of adverse events as a result of LLOM choice of therapy, such as new need for renal replacement therapy; an elevated creatine kinase level (ie, an increase of >500 U/L from baseline 30 d after antibiotic initiation); a vancomycin infusion reaction (ie, documented flushing, pruritus, hypotension, chest pain, or dyspnea during infusion); *Clostridioides difficile* within 90 days of treatment; and drug-induced immune thrombocytopenia (>150,000 platelet count per mcL decrease from baseline within 30 d of antibiotic initiation). Other secondary outcomes included hospital readmission for LLOM within 180 days of antibiotic completion and hospital length of stay.

### Statistical analysis

Descriptive statistics measured baseline characteristics, imaging, and culture results. Descriptive analysis was performed on all continuous variables. Mean ± standard deviation was used to represent normally distributed variables, and median ± interquartile range was used for nonnormally distributed variables. Continuous data were analyzed using the student’s t-test or Mann-Whitney U test as appropriate. Count and proportions were used for all categorical variables. The categorical variables were analyzed using chi-square test or Fisher’s exact test as appropriate. Variables identified in univariate analysis as being associated with clinical failure with p-values <0.20 were entered into a multivariable logistic regression analysis. P-values <0.05 were considered statistically significant.

## Results

### Study patients and baseline characteristics

A total of 473 patients were admitted with an LLOM within Methodist Health System between April 1, 2017, and April 1, 2023 (Figure 1). Of these, 64 were included for analysis (Table 1). The median (IQR) age of the patients was 61 (53–71) years and most were male (75%), with a median (IQR) serum creatinine level of 1 (0.8–2.04) mg/dL and median (IQR) Charles comorbidity index score of 5.5 (3–9). Several of the patients had chronic comorbidities, including diabetes (68.8%), end-stage renal disease on hemodialysis (17.2%), chronic kidney disease (42.2%), and/or a prior amputation (21.9%). Forty-six (71.9%) patients had a pathogen identified on their cultures.

**Table 1.** Baseline characteristics of the study cohort

Variables	Total (N = 64)	AMT (N = 26)	NAMT (N = 38)	P-value
Age (years), median (IQR)	60 (53–71)	61 (53.4–74)	60 (54–71)	0.712
Gender (male), n (%)	48 (75)	20 (77)	28 (73.7)	0.7688
SCr on admission (mg/dL), median (IQR)	1.07 (0.8–2.04)	1 (0.78–2.1)	1.1 (0.8–1.9)	0.404
Charlson Comorbidity Index score, median (IQR)	6 (3–9)	5 (3–9)	6 (4–9)	0.028
Infectious disease consultation, n (%)	60 (93.8)	24 (92.3)	36 (94.7)	0.539
**Allergy History^a^, n (%)**				
Penicillin (piperacillin/tazobactam, amoxicillin/clavulanate)	8 (13)	3 (12)	5 (13)	
Cephalosporin (cephalexin, cefdinir)	4 (6)	0 (0)	4 (11)	
Doxycycline	2 (3)	0 (0)	2 (5)	
Macrolide (azithromycin, erythromycin)	2 (3)	1 (4)	1 (3)	
Sulfamethoxazole/trimethoprim	3 (5)	1 (4)	2 (5)	
Aminoglycoside (tobramycin, gentamicin)	2 (3)	0 (0)	2 (5)	
Nitrofurantoin	1 (2)	1 (4)	0 (0)	
Levofloxacin	1 (2)	0 (0)	1 (3)	
Meropenem	1 (2)	0 (0)	1 (3)	
Linezolid	1 (2)	0 (0)	1 (3)	
Clindamycin	2 (3)	1 (4)	1 (3)	
Metronidazole	1 (2)	0 (0)	1 (3)	
**Comorbidities^b^, n (%)**				
Prior amputations	14 (21.9)	6 (23.1)	8 (21.1)	0.076
Peripheral arterial disease	4 (6.3)	0 (0)	4 (10.5)	0.124
Diabetes mellitus	44 (68.8)	18 (72)	26 (68.4)	0.092
History of MRSA infection at current site	1 (1.6)	0 (0)	1 (2.7)	0.597
Retained hardware	2 (3.1)	0 (0)	2 (5.3)	0.349
Congestive heart failure	18 (28.1)	6 (23.1)	12 (31.6)	0.552
Chronic kidney disease	27 (42.2)	12 (46.2)	15 (39.5)	0.282
ESRD on HD	11 (17.2)	4 (15.4)	7 (18.4)	0.514
Chronic obstructive pulmonary disease	13 (20.3)	5 (19.2)	8 (21.1)	0.032
Connective tissue disorder	3 (4.7)	0 (0)	3 (7.9)	0.202
Cerebral vascular accident	8 (12.5)	3 (11.5)	5 (13.2)	0.583
Coronary artery disease	7 (10.9)	2 (7.7)	5 (13.2)	0.398
Dementia	9 (14.1)	5 (19.2)	4 (10.5)	0.266
Hemiplegia	5 (7.9)	1 (3.8)	4 (10.5)	0.318
Peripheral vascular disease	28 (43.8)	9 (34.6)	19 (50)	1.485
Liver disease	3 (4.7)	0 (0)	3 (7.9)	0.349
Peptic ulcer disease	4 (6.3)	2 (7.7)	2 (5.3)	0.539
Rheumatoid arthritis	2 (3.1)	0 (0)	2 (5.3)	0.51
**Pathogen distribution^c^, n (%)**				
**Identified pathogen^d^**	46 (72)	13 (50)	33 (87)	
MSSA	12 (19)	2 (8)	10 (26)	
*Streptococcus* species	10 (16)	2 (8)	8 (21)	
*Pseudomonas* species	6 (9)	1 (4)	5 (13)	
**No pathogen identified**	18 (28)	13 (50)	5 (13)	

Abbreviations: AMT, anti-MRSA therapy; ESRD, end-stage renal disease; HD, hemodialysis; MRSA, methicillin-resistant *Staphylococcus aureus*; MSSA, Methicillin-susceptible *Staphylococcus aureus*; NAMT, no-anti-MRSA therapy; SCr, serum creatinine.

a Multiple allergies possible for individual patients.

b Multiple comorbidities possible for individual patients.

c Multiple pathogens possible for individual patients.

d Other pathogens identified: *Staphylococcus epidermidis*, *Providencia stuartii, Enterococcus faecalis, Enterobacter cloacae, Proteus mirabilis, Peptostreptococcus anaerobius, Aspergillus niger, Staphylococcus simulans, Micromonas.*

Of the 64 patients included in analysis, 26 (40.6%) received AMT and 38 (59.4%) received no-anti-MRSA therapy (NAMT). The most common therapies used in the AMT group were vancomycin (65%), daptomycin (23%), doxycycline (8%), and dalbavancin (4%) (Table 2). Most baseline characteristics were comparable between the AMT and NAMT treatment arms (Table 1). However, the NAMT cohort showed higher incidence of hemiplegia (10.5% vs 3.8%, *P* = 0.318), history of MRSA at current site of infection (2.7% vs 0%, *P* = 0.597) and retained hardware (5.3% vs 0%, *P* = 0.349); these differences were not statistically significant. Thirteen (50%) in the AMT group and 37 (86.8%) patients in the NAMT group had a pathogen identified on cultures. The pathogens most prevalent were MSSA (8% AMT vs 26% NAMT), followed by *Streptococcus* species (8% AMT vs 21% NAMT) and *Pseudomonas* species (4% AMT vs 13% NAMT). The only statistically significant difference between the cohorts was the Charles comorbidity index score (5 AMT vs 6 NAMT, *P* = 0.028).

**Table 2.** Antibiotic utilization

Antibiotic^a^, n (%)	AMT (N = 26)	NAMT (N = 38)
Dalbavancin	1 (4)	0 (0)
Daptomycin^b^	6 (23)	0 (0)
Doxycycline^b^	2 (8)	0 (0)
Vancomycin	17 (65)	0 (0)
Ampicillin	0 (0)	1 (3)
Ampicillin/sulbactam	1 (4)	0 (0)
Cefazolin	0 (0)	6 (16)
Cefepime	6 (23)	3 (8)
Ceftazidime	1 (4)	0 (0)
Ceftriaxone	5 (19)	16 (42)
Ciprofloxacin^b^	1 (4)	2 (5)
Ertapenem	4 (15)	2 (5)
Fluconazole^b^	0 (0)	1 (3)
Meropenem	4 (15)	3 (8)
Metronidazole^b^	3 (12)	7 (18)
Piperacillin/tazobactam	1 (4)	5 (13)

Abbreviations: AMT, anti-MRSA therapy; NAMT, no-anti-MRSA therapy.

a Multiple antibiotics could be used for individual patients.

b Combined with IV therapy if given by mouth.

### Patient outcomes

In the total cohort, 18 (28%) patients had treatment failure (Table 3). Both treatment arms were comparable in treatment failure subgroups: repeat antibiotic treatment within 180 days of completion (7.7% AMT vs 18.4% NAMT, *P* = 0.291), unplanned surgical intervention during index admission after completing ≥4 weeks of therapy (7.7% AMT vs 10.5% NAMT, *P* = 0.531), and death by any cause during index admission (11.5% AMT vs 15.8% NAMT, *P* = 0.728). A composite of the treatment failure subgroups above revealed 6 (23%) patients in the AMT group and 12 (32%) patients in the NAMT group failed therapy (*P* = 0.325). The biggest contributor to AMT’s treatment failure was death from any cause. For the NAMT group, it was from the need to repeat antibiotics within 180 days of completion.

**Table 3.** Patient outcomes

	Total (N = 64)	AMT (N = 26)	NAMT (N = 38)	P-value
**Primary Outcomes, n (%)**				
Treatment failure	18 (28.1)	6 (23.1)	12 (31.6)	0.325
Repeat antibiotic treatment within 180 days of completion	9 (14.1)	2 (7.7)	7 (18.4)	0.291
Unplanned surgical intervention during index admission after completing ≥4 weeks of therapy	6 (9.4)	2 (7.7)	4 (10.5)	0.531
Death by any cause during index admission	9 (14.1)	3 (11.5)	6 (15.8)	0.728
**Secondary Outcomes**				
Hospital readmission within 180 days of antibiotic completion, n (%)	30 (46.9)	12 (46.2)	18 (47.4)	0.92
Hospital length of stay (days), median (IQR)	7 (5–13)	6 (5–9)	7 (5–12.5)	0.285
Adverse events, n (%)				
RRT	1 (1.6)	0 (0)	1 (2.6)	0.594
Elevated CK	1 (1.6)	0 (0)	1 (2.6)	0.594
DITP	2 (3.1)	0 (0)	2 (5.3)	0.510

Abbreviations: RRT, renal replacement therapy; AMT, anti-MRSA therapy; CK, creatine kinase; DITP, drug-induced immune thrombocytopenia; NAMT, no-anti-MRSA therapy.

The following adverse events were observed in the cohort: new need for renal replacement therapy (0% AMT vs 2.6% NAMT, *P* = 0.594), elevated creatine kinase level (0% AMT vs 2.6% NAMT, *P* = 0.594), and drug-induced immune thrombocytopenia (0% AMT vs 5.3% NAMT, *P* = 0.349). No patients experienced vancomycin infusion syndrome or *Clostridioides difficile* infection. Overall, there were no statistically significant differences in patient outcomes between the AMT and NAMT treatment arms.

## Discussion

In this study, patients with and without AMT for the treatment of LLOM with cultures negative for MRSA were evaluated. The composite primary outcome of treatment failure was not found to be statistically different between groups. Numerically more patients met each of the individual components of treatment failure (ie, repeat antibiotics, unplanned surgery, or death) in the NAMT group, with the repeat antibiotic utilization being most notable, although no differences were statistically significant. Secondary clinical outcomes of hospital length-of-stay and readmission were similar between groups. Although we hypothesized the AMT group would have an increased incidence of adverse events, our results revealed that safety and efficacy were comparable between the AMT and NAMT treatment arms.

The lack of difference in adverse events between AMT and NAMT groups was unexpected. Adverse events occurred at low rates and given small sample size of the study it is difficult to discern any true disparity. Still that adverse events occurred only in the NAMT group is unexpected. This may be explained by the adverse events being unrelated to the antimicrobial therapy. The elevated CK observed in a patient receiving NAMT exemplifies this as there is no currently reported connection known with the treatment antibiotic (meropenem), and it did not lead to a change in therapy by the treatment team. The other adverse events observed may also be unrelated to the antibiotic therapy further demonstrating the challenges of evaluating adverse effects of drug therapy in retrospective studies.

With balanced treatment arms, this study did well to reflect the common LLOM findings addressed in primary literature. Barshes and colleagues evaluated the rate of treatment failure and leg amputations among patients with foot OM.^14^ In 184 cases, 53 (28%) patients had treatment failure, and 21 (11.4%) patients had a leg amputation. Major risk factors that correlated with poor outcomes included peripheral arterial disease, homelessness, *Pseudomonas aeruginosa* or *Escherichia coli* bone isolates, a serum albumin level <2.8 mg/dL, hallux involvement, insulin therapy, smoking history of ≥60 pack-years, and <7 days of antibiotic therapy for a positive bone margin. In addition, after comparing our findings to those in primary literature, our rate of treatment failure for all patients, regardless of the treatment, is comparable (28%). This further validates our chosen sample. Overall, the study did well to point out the frequency of treatment failure with OM and the importance of covering pathogens appropriately.

Our study addressed the clinical question of whether AMT is beneficial to patients with LLOM with cultures negative for MRSA, despite guideline recommendations. To our knowledge, this is the first study that focused on clinical outcomes of AMT in this population. However, there have been studies that have evaluated the frequency of this practice. One retrospective cohort study evaluated the concordance of empiric antibiotic therapy, microbiologic results, and definitive therapy to positive MRSA or resistant gram-negative organisms in 259 patients with DFI and LLOM.^15^ Of the 259 patients, 234 (90.3%) had microbiological testing performed. Ninety-one (35%) out of 224 patients on empiric AMT were discharged on definitive therapy against MRSA but only 29 (12.4%) out of 234 patients had a positive culture for MRSA. Together, this indicates 62 (24%) out of the 91 patients were on AMT unnecessarily. However, it was unclear if patients included in the AMT group completely overlapped with the patients in the group with cultures drawn. Therefore, there were 25 patients who did not have cultures drawn but could have had a MRSA infection. Regardless, there would still be an excess number of patients on AMT needlessly.

Reveles and colleagues performed a retrospective cohort study that looked at AMT prevalence and appropriateness.^16^ Of 318 culture-positive DFI patients, 15% grew MRSA. In total, 273 (86%) patients received AMT, which meant AMT was dispersed unnecessarily in 71% of the cases. This study and the one prior give just a glimpse of how common AMT is among patients with cultures negative for MRSA.

It is unclear why the practice of supplying AMT unnecessarily continues. Possibly, providers consider this a better option than narrowing therapy to guarantee resistant pathogens are covered appropriately. However, the Infectious Diseases Society of America and other healthcare organizations recommend narrowing antibiotic therapy as quickly as possible. Therapies such as vancomycin, daptomycin or dalbavancin require close clinical monitoring because they are associated with adverse drug events such as acute kidney injury, thrombocytopenia, infusion reactions, myopathy, subsequent infections, and possible microbial resistance. A strength of our study was that it focused on examining whether AMT in patients with cultures negative for MRSA and LLOM is advantageous over narrow therapy.

Limitations of the study includes the small sample size, as we had only 64 patients to evaluate after applying the exclusion criteria. The small sample size can minimize generalizability to other practice sites and lead to the potential for type II error. In the future, a larger sample size can be examined to definitively determine if there is a difference in outcomes between the two treatment arms. A subgroup analysis to evaluate patients with and without a pathogen grown on cultures would be beneficial to evaluate in the future as well. Unfortunately, our creatinine kinase measure was not well defined since patients with elevated levels prior to admission were not excluded. Leading to one person having an elevated CK in the NAMT group. Another limitation is that because of the retrospective design of this study, analysis of patient cases were limited to chart review, making safety end points such as cause of death and adverse events difficult to confirm.

In conclusion, this study revealed there were no clinically significant differences in the efficacy or safety of using AMT or NAMT in those with cultures negative for MRSA. A larger study of this clinical scenario with well-defined inclusion/exclusion criteria and clear outcome parameters would be valuable in evaluating the risk factors, predictors of clinical failure, and epidemiology of MRSA in LLOM. Such study would also improve prediction of clinical failure, patient care outcomes overall, and optimize outcomes with antibiotic usage.

## Research transparency and reproducibility

No additional data to report.
